# Dynamic Behavior for an SIRS Model with Nonlinear Incidence Rate and Treatment

**DOI:** 10.1155/2013/209256

**Published:** 2013-11-21

**Authors:** Junhong Li, Ning Cui

**Affiliations:** Department of Mathematics and Sciences, Hebei Institute of Architecture & Civil Engineering, Zhangjiakou, Hebei 075000, China

## Abstract

This paper considers an SIRS model with nonlinear incidence rate and treatment. It is assumed that susceptible and infectious individuals have constant immigration rates. We investigate the existence of equilibrium and prove the global asymptotical stable results of the endemic equilibrium. We then obtained that the model undergoes a Hopf bifurcation and existences a limit cycle. Some numerical simulations are given to illustrate the analytical results.

## 1. Introduction

Treatment is an important and effective method to prevent and control the spread of various infectious diseases, such as measles, tuberculosis, and flu [1–4]. In classical epidemic models, the treatment rate of the infective is assumed to be proportional to the number of the infective individuals [5]. This is unsatisfactory because the resources for treatment should be quite large. In fact, every community should have a suitable capacity for treatment. If it is too large, the community pays for unnecessary cost. If it is small, the community has the risk of the outbreak of a disease. Thus, it is realistic to maintain a suitable capacity of disease treatment. Wang and Ruan [6] considered an SIR model in which the capacity for the treatment of a disease in a community is a constant. Namely, they used the following function:

(1) $$\begin{array}{c} h\left(I\right)=\{\begin{array}{cc} k, & \text{if}\;\;I>0,\\ 0, & \text{if}\;\;I=0, \end{array} \end{array}$$

which was used by [7]. This seems more reasonable when we consider the limitation of the treatment resource of a community. 

There are many reasons for using nonlinear incidence rate, and various forms of nonlinear incidence rates have been proposed recently. Liu et al. [8] proposed a nonlinear saturated incidence function *g*(*I*)*S* = *βSI* ^*p*^/(1 + *αI* ^*q*^) to model the effect of behavioral changes to certain communicable disease, where *βSI* ^*p*^ describes the infection force of the disease and 1/(1 + *αI* ^*q*^) measures the inhibition effect from the behavioral change of the susceptible individuals when the number of infectious individuals increases. The case when *p* = 1, *q* = 2 was used by [9]. We assume the population can be partitioned into three compartments: susceptible, infective, and recovered. Let *S*, *I*, and *R* denote the numbers of susceptible, infective, and recovered individuals, respectively. Motivated by the works [6, 7, 9], we will formulate an SIRS model with nonlinear incidence rate and constant immigration rates for susceptible and infectious individuals [10]. Namely, we consider the following SIRS model:

(2) $$\begin{array}{c} S^{\prime }=\left(1-p\right)A-\frac{\beta IS}{1+\alpha I^{2}}-dS+\gamma R,\\ R^{\prime }=mI-\left(d+\gamma \right)R+h\left(I\right),\\ I^{\prime }=pA+\frac{\beta IS}{1+\alpha I^{2}}-\left(d+m\right)I-h\left(I\right), \end{array}$$

where *d* is the rate of natural death, *m* is the rate for recovery, *β* is the proportionality constant, *γ* is the rate at which recovered individuals lose immunity and return to susceptible class, *α* is the parameter measures of the psychological or inhibitory effect, and (1 − *p*)*A*, *pA* are constant recruitments of susceptible and infective individuals, respectively. It is assumed that all the parameters are positive constants. It is easy to see that the total population size *N* implies *N*′ = *A* − *dN*. Since *N* tends to a constant as *t* tends to infinity, we assume that the population is in equilibrium and investigate the behavior of (2) on the plane *S* + *I* + *R* = *A*/*d* = *N*_0_ > 0. Let (*S*(*t*), *I*(*t*), *R*(*t*)) be a solution of (2) with initial conditions *S*(0) ≥ 0, *I*(0) ≥ 0, and *R*(0) ≥ 0. This solution will satisfy *S*(*t*) ≥ 0, *I*(*t*) ≥ 0, and *R*(*t*) ≥ 0 for all *t* ≥ 0 since *S*′ = (1 − *p*)*A* + *γR* > 0 if  (0, *I*(*t*), *R*(*t*)) ∈ *R*_+_^3^, *I*′ = *pA* > 0 if (*S*(*t*), 0, *R*(*t*)) ∈ *R*_+_^3^, and *R*′ = *mI* + *h*(*I*) ≥ 0 if (*S*(*t*), *I*(*t*), 0) ∈ *R*_+_^3^. Consequently, *R*_+_^3^ is positively invariant for (2). Thus, we restrict our attention to the following reduced model:

(3) $$\begin{array}{c} I^{\prime }=pA-k+\frac{\beta I\left(N_{0}-I-R\right)}{1+\alpha I^{2}}-\left(d+m\right)I,\\ R^{\prime }=mI-\left(d+\gamma \right)R+k, \end{array}$$

where *k* is the treatment constant.

From the epidemiological interpretation, our discussion on (3) will be restricted in the following bounded domain:

(4) $$\begin{array}{c} D=\left\{\left(I,R\right):I>0,\;\;R>0,\;\;0<I+R<N_{0}\right\} \end{array}$$

which is a positively invariant set for (3).

The paper is organized as follows. In the next section, we investigate the existence and stability of equilibrium for (3). In Section 3, we study the Hopf bifurcation and limit cycle. Some numerical simulations are given to illustrate the analytical results.  Section 4 is a brief discussion.

## 2. Existence and Stability of Equilibrium

In this section, we first consider the existence of equilibrium of (3) and their global stability. In order to find endemic equilibrium of (3), we substitute

(5) $$\begin{array}{c} R=\frac{mI+k}{d+\gamma } \end{array}$$

into

(6) $$\begin{array}{c} pA-k+\frac{\beta I\left(N_{0}-I-R\right)}{1+\alpha I^{2}}=\left(d+m\right)I \end{array}$$

to obtain the cubic equation

(7) f(I)=α(d+m)I3+[β(d+γ+m)d+γ+(k−pA)α]I2 +[d+m+βkd+γ−βN0]I+k−pA=0.

Let *I*_1_, *I*_2_, and *I*_3_ be three roots of (7). Then, we get

(8) $$\begin{array}{c} \frac{\beta }{\alpha \left(d+m\right)}\left(\frac{A}{d}-\frac{k}{d+\gamma }\right)=\frac{1}{\alpha }-\left(I_{1}I_{2}+I_{1}I_{3}+I_{3}I_{2}\right),\\ \frac{\beta }{\alpha \left(d+m\right)}\left(1+\frac{m}{d+\gamma }\right)=I_{1}I_{2}I_{3}\alpha -\left(I_{1}+I_{2}+I_{3}\right). \end{array}$$

When *R*_0_ = *k*/*pA* < 1, we can see that

(9) $$\begin{array}{c} \frac{I_{1}+I_{2}+I_{3}}{I_{1}I_{2}I_{3}}<\alpha <\frac{1}{I_{1}I_{2}+I_{2}I_{3}+I_{1}I_{3}}; \end{array}$$

then there is a unique positive root of (7). Direct calculations show that

(10) f(N0)=(1−p)B+γN0+N02[βγN0(d+ε)+γαN0+(1−p)dαN0]>0,f(pA−kd+m)=βpA(1−R0)(d+m)2(d+γ) ×[−kγ+N0(p−1)d(d+γ+m)−N0γm] <0 if  R0<1.

From biological considerations, it is easy to see the positive root

(11) $$\begin{array}{c} I\in \left(\frac{pA-k}{d+m},N_{0}\right). \end{array}$$

Based on the above analysis, we obtain the following theorem.

**Theorem 1**

There is a unique endemic equilibrium *E*_0_(*I*_*e*_, *R*_*e*_) of (3) if *R*_0_ < 1.

**Theorem 2**

The endemic equilibrium *E*_0_ of (3) is locally asymptotically stable if *R*_0_ < 1.

**Proof**

The Jacobian matrix of (3) at *E*_0_ takes the form

(12) J(E0)= [pA(R0−1)Ie−2αβIe2[N0−Ie−Re](1+αIe2)2−βIe1+αIe2−βIe1+αIe2m−d−γ].

It is easy to obtain tr⁡(*J*(*E*_0_)) < 0 and det⁡(*J*(*E*_0_)) > 0 when *R*_0_ < 1. This completes the proof.

**Theorem 3**

The endemic equilibrium *E*_0_ of (3) is globally asymptotically stable if *R*_0_ < 1.

**Proof**

Taking Dulac function

(13) $$\begin{array}{c} D=\frac{1+\alpha I^{2}}{I}, \end{array}$$

we obtain

(14) ∂(PD)∂I+∂(QD)∂R=−β−2α(d+m)I−pA(1−R0)I2 +αpA(1−R0)−d+γI−αI(d+γ)≤−β−αpA(1−R0)I −pA(1−R0)I2−d+γI−αI(d+γ),

where (*P*, *Q*) is the vector field of (3). Obviously,

(15) $$\begin{array}{c} \frac{\partial \left(PD\right)}{\partial I}+\frac{\partial \left(QD\right)}{\partial R}<0\;\text{if}\;\;R_{0}<1. \end{array}$$

Then by Dulac's criteria, the system (3) admits no limit cycles or separatrix cycles. The global stability of *E*_0_ follows from the Poincare-Bendixson Theorem. This completes the proof.

**Theorem 4**

There will be one or two endemic equilibria *E*_∗_(*I*_∗_, *R*_∗_) of the system (3) if *R*_0_ > 1.

**Proof**

Based on the analysis of Theorem 1, when *R*_0_ > 1, we obtain the roots of (7) satisfying

(16) $$\begin{array}{c} I_{1}+I_{2}+I_{3}=-\frac{\beta \left(d+\gamma +m\right)+\alpha pA\left(R_{0}-1\right)\left(d+\gamma \right)}{\alpha \left(d+m\right)\left(d+\gamma \right)}<0,\\ I_{1}I_{2}I_{3}=-\frac{pA\left(R_{0}-1\right)}{\alpha \left(d+m\right)}<0; \end{array}$$

then there exist one positive real root and two conjugate complex roots with negative real parts or two positive real roots and one negative root. Thus, there will be one or two endemic equilibria in (3). This completes the proof.

## 3. Hopf Bifurcation

In this section, we study the Hopf bifurcation and limit cycle of the system (3). For simplicity of computation, we consider the following system which is equivalent to (3):

(17) I′=βI(N0−I−R)−(d+m)I(1+αI2) +(1−R0)pA(1+αI2),R′=[mI−(d+γ)R+pAR0](1+αI2).

Let *x* = *I* − *I*_∗_, *y* = *R* − *R*_∗_ to translate *E*_∗_ to (0,0). Then, (17) becomes

(18) $$\begin{array}{c} x^{\prime }=a_{11}x+a_{12}y+f_{1}\left(x,y\right),\\ y^{\prime }=a_{21}x+a_{22}y+f_{2}\left(x,y\right), \end{array}$$

where *f*_1_(*x*, *y*) and *f*_2_(*x*, *y*) represent the higher order terms and

(19) a11=β(N0−2I∗−R∗)−(d+m)(1+3αI∗2) −2pAα(R0−1)I∗,a12=−βI∗,  a21=m(1+αI∗2),  a22=−(d+γ)(1+αI∗2).

To obtain the Hopf bifurcation, we fix parameters such that tr⁡(*J*(*E*_∗_)) = 0, which is equivalent to *a*_11_ = (*d* + *γ*)(1 + *αI*_∗_^2^). Let

(20) $$\begin{array}{c} X=x,\;\;Y=a_{11}x+a_{12}y; \end{array}$$

then (18) is reduced to

(21) X′=Y+f1(X,Y−a11Xa12),Y′=−δX+a11f1(X,Y−a11Xa12)+a12f2(X,Y−a11Xa12),

where

(22) δ=(d+r)2(1+αI∗2)2−mβI∗(1+αI∗2)>0if  (d+r)2(1+αI∗2)>mβI∗.

Let

(23) $$\begin{array}{c} u=-X,\;\;v=\frac{Y}{\sqrt{\delta }}; \end{array}$$

we obtain the normal form of the Hopf bifurcation:

(24) $$\begin{array}{c} u^{\prime }=-\sqrt{\delta }v+F_{1}\left(u,v\right),\\ v^{\prime }=\sqrt{\delta }u+F_{2}\left(u,v\right), \end{array}$$

where

(25) F1(u,v)=δuI∗−(d+m)αu3 −[β+3dαI∗+3mαI∗−pAα(R0−1)   −(d+α)(1+αI∗2)I∗]u2,

(26) F2(u,v)=1δ[−mα+α(d+γ)2(1+αI∗2)βI∗]u3 +1δ[2mαI∗−2α(d+γ)2(1+αI∗2)β] +α(d+γ)u2vβI∗−m(1+αI∗2)δ −2α(d+γ)uv2β+(d+γ)(1+αI∗2)vδβI∗.

Set the Lyapunov number by

(27) σ=116[∂3F1∂u3+∂3F1∂u∂v2+∂3F2∂u2∂v+∂3F2∂v3    +∂2F1∂u∂v(∂2F1∂u2+∂2F1∂v2)−∂2F2∂u∂v(∂2F2∂u2+∂2F2∂v2)    −∂2F1∂u2∂2F2∂u2+∂2F1∂v2∂2F2∂v2],

which can be reduced to

(28) $$\begin{array}{c} \sigma =\frac{1}{16}\left[\frac{2\alpha \left(d+\gamma \right)}{\beta I_{*}}-6\alpha \left(d+m\right)\right]. \end{array}$$

So we have the following Hopf bifurcation results.

**Theorem 5**

There exist Hopf bifurcation and limit cycle in the system (17), when

(29) $$\begin{array}{c} a_{11}=\left(d+\gamma \right)\left(1+\alpha I_{*}^{2}\right),\;{\left(d+r\right)}^{2}\left(1+\alpha I_{*}^{2}\right)>m\beta I_{*}. \end{array}$$

To illustrate the theorem, let us consider the following parameters.

*β* = 0.01 (see [11]), *m* = 0.1 (see [12]), *A* = 4.4236, *d* = *k* = *γ* = 0.1, *α* = 0.3995, and *p* = 0.01.

**Figure 1:**
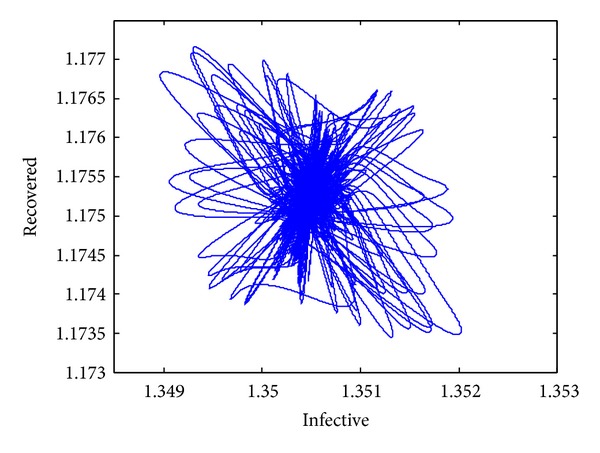
The phase portraits of (17).

**Figure 2:**
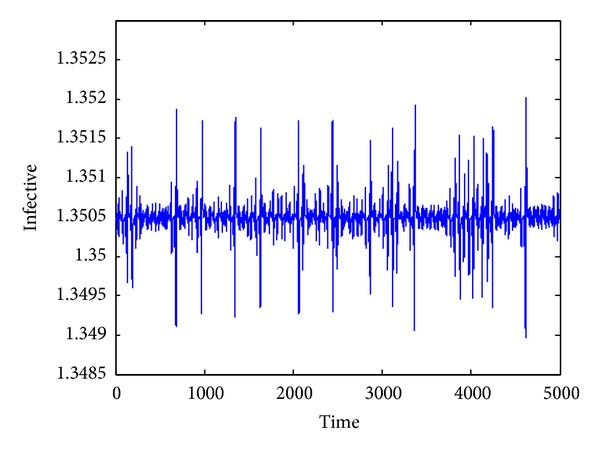
Time series of infective individuals.

**Figure 3:**
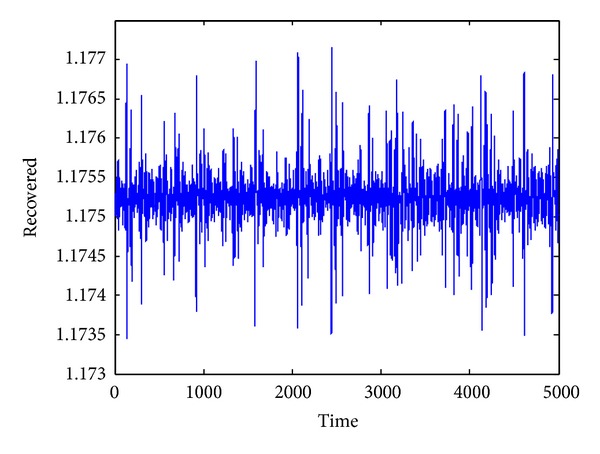
Time series of recovered individuals.

We have *R*_0_ = 2.2606 > 1, and the equilibria *E*_1_(0.25,0.625), *E*_2_(1.3505,1.1752) exist (see Theorem 4). The equilibrium *E*_2_ is unstable saddle. The parameter values satisfy conditions (29) of Theorem 5 and *σ* = 3.965. Therefore, (17) has an unstable periodic orbit which encircles *E*_1_. Its phase portrait is illustrated in Figure 1. The time series of the infective and recovered individuals are given in Figures 2 and 3, respectively.

## 4. Conclusion

In this paper, we discuss an SIRS epidemic model with nonlinear incidence rate and treatment. It is assumed that susceptible and infectious individuals have constant immigration rates. We investigate the existence and stability of equilibria of (3) and study the Hopf bifurcation and limit cycle. Some numerical simulations are given to illustrate the analytical results. Without the treatment and recruitment of infectious, (2) becomes the SIRS model (see [9]).
